# Are Price Limits Effective? An Examination of an Artificial Stock Market

**DOI:** 10.1371/journal.pone.0160406

**Published:** 2016-08-11

**Authors:** Xiaotao Zhang, Jing Ping, Tao Zhu, Yuelei Li, Xiong Xiong

**Affiliations:** 1 College of Management and Economics, Tianjin University, Tianjin, P.R. China; 2 Key Laboratory of Computation and Analytics of Complex Management Systems (CACMS), Tianjin, P.R. China; 3 Research Institute, Bohai Securities Co., Ltd, Tianjin, P.R. China; East China University of Science and Technology, CHINA

## Abstract

We investigated the inter-day effects of price limits policies that are employed in agent-based simulations. To isolate the impact of price limits from the impact of other factors, we built an artificial stock market with higher frequency price limits hitting. The trading mechanisms in this market are the same as the trading mechanisms in China’s stock market. Then, we designed a series of simulations with and without price limits policy. The results of these simulations demonstrate that both upper and lower price limits can cause a volatility spillover effect and a trading interference effect. The process of price discovery will be delayed if upper price limits are imposed on a stock market; however, this phenomenon does not occur when lower price limits are imposed.

## 1 Introduction

The establishment of price limits is one of the most widely used price stabilization mechanisms in the stock market, especially in emerging markets such as China’s stock market. However, there are always controversies regarding price limits mechanisms. Proponents believe that price limits can provide sufficient time to identify market information and re-evaluate the intrinsic value of stocks. Ma et al. [1] argue that price limits can provide a cooling-off period for the market and allow traders the time to digest the causes of the substantial price revisions that culminate in the activation of the limits. Huang et al.’s [2] conclusion is consistent with the overreaction hypothesis, where an overreaction is delayed and corrected by price limits; thus, the results support the validity of price limits. Kim et al. [3] studied the price limits policy in China’s stock market by comparing a sub-period with price limits with a different sub-period without price limits; these researchers suggest that price limits can decrease short-term volatility and mitigate abnormal trading activity. However, the sub-period without price limits in their study was the early time of China’s stock market. At this time, the market was very small with only a dozen listed stocks, investors could not distinguish between stocks and bonds, and their opportunities to learn about trading mechanisms were limited. The question regarding whether these results can contribute to a mature market has not yet been considered.

Opponents believe that price limits will hinder market information transmission, increase information asymmetry, delay the process of price discovery and reduce market efficiency. Opponents also focus on three hypotheses, namely, the volatility spillover hypothesis, the delayed price discovery hypothesis and the trading interference hypothesis. Kim and Rhee [4] empirically tested these hypotheses regarding the effects of price limits on the Tokyo Stock Exchange, and they compared stock volatility, trading volume and returns among all groups of stocks. Their findings support each of the three hypotheses and suggest that price limits may be ineffective. These findings are also supported by Kim and Limpaphayom [5]. Using Kim and Rhee (1997)’s empirical method, Qu [6] researched the effects of price limits on China’s stock market. Qu confirmed the efficacy of the three hypotheses based on the existence of short-selling restrictions and concluded that widening price limits would have a greater impact on market efficiency. Yeh and Yang [7] found evidence of delayed price discovery and trading interference, and the significance of these phenomena depends on the level of the price limits. Although Chang and Hsieh [8] found no evidence of the volatility spillover hypothesis and the trading interference hypothesis, they determined that the process of delayed price discovery exists. Investors are more likely to purchase stocks that hit upper limits “at a high price” than sell stocks that hit lower limits “at a low price.” Wang et al. [9] arrived at the same conclusion.

The original purpose of setting price limits was to prevent irrational price fluctuations and minimize investor losses. Tian and Cao [10] confirmed that price limits can decrease the volatility of the Chinese stock market. Furthermore, Chen et al. [11] found that the effect of price limits is asymmetric for bullish and bearish sample periods. During a bullish period, price limits effectively reduce the stock volatility for downward price movements, not for upward price movements, whereas during a bearish period, price limits effectively reduce the stock volatility for upward price movements, not for downward price movements. However, contradictory conclusions have also been found. Kim and Yang [12] considered that price limits do not play a role in reducing volatility, and they even concluded that price limits will increase market volatility and lead to market overreaction.

Concerning liquidity, which is affected by price limits, scholars have different opinions. Fama [13] argues that price limits do not halt trading but prevent immediate corrections in order imbalance. The liquidity of a stock is restricted when the stock hits the price limits, which causes an increase in liquidity on the following trading days, as discussed by Zhuang and Zhao [14]. Price limits have different liquidity effects on different types of stock, which is obvious in the cases of stocks with a higher systematic risk, market value, turnover rate and PE ratio [15].

According to the above findings, researchers have not reached consistent conclusions regarding what and how price limits affect stock markets, but this trading mechanism clearly has a significant effect on stock price behavior [16]. However, most of the relevant reports in the literature have involved examinations of price limits using empirical methods and event studies, and various conclusions have been obtained for different regional markets and time periods. It is difficult to find a benchmark for a specialized market in which price limits are altered but the remaining trading mechanisms remain unchanged because there are significant costs and risks associated with changing trading mechanisms in actual markets. Agent-based computational finance (ACF) provides a simple and feasible means of solving this problem. Lux and Marchesi [17] demonstrated that an agent-based model can generate behaviors that arise from mutual interactions among participants in actual financial markets. An artificial stock market should simulate market structure, trading mechanism and price formation. Mike and Farmer [18] constructed an empirical behavioral model (the MF model) based on the continuous double auction mechanism in an order-driven market to simulate the dynamic process of stock price formation. Subsequently, Gu and Zhou [19] modified the MF model by incorporating long memory into the aggressiveness of incoming orders. The return distributions of mock stocks in the MF model and the modified MF model have also been examined [20,21]. ACF has been used to study transaction taxes [22–24]; order books and order flows [25]; regulatory policies [26]; and flash crashes [27]. Westerhoff [26], who is commonly regarded as the first scholar to use agent-based modeling to examine the effectiveness of price limits, found that price limits can reduce volatility and decrease price distortion if the limits are not overly restrictive. Yeh and Yang [7,28] reached a similar conclusion. However, the models used in these studies only allow agents to place orders of unit size and cannot incorporate feedback associated with the ongoing evolution of the market. To offset these disadvantages, Chiarella et al. [25,29] proposed a model of an order-driven market in which traders set bids, determine ask prices, conduct post-market trading, and submit limits orders according to exogenously fixed rules. In addition, Chiarella et al. found that large price changes are likely to be generated by the presence of large gaps in the books. Li et al. [30] modeled an artificial stock market with trading mechanisms similar to those of the real Chinese stock market; these researchers promoted a scaling calibration method. Furthermore, the volatility of China’s stock market is clearly higher than the volatilities of developed markets [31]. Accordingly, we used the models of Chiarella et al. [25] and Li et al. [30] to establish an artificial stock market with trading mechanisms that simulates China’s stock market. In the present article, a combination of the agent-based method and the empirical approach used by Kim and Rhee [4] is utilized to study the inter-day effects caused by price limits, and the ways in which price limits affect market efficiency are discussed.

## 2 The Artificial Stock Market

In this section, the approach used by Chiarella et al. [25], Chiarella and Iori [29] and Li et al. [30] is employed to build an order-driven stock market with a boundedly rational heterogeneous agent that is modified in accordance with the Chinese stock market’s trading mechanisms and regulations.

### 2.1 Agent decision process

The model assumes that there are two assets in the market: one is a risk-free asset, such as cash, and the other is a high-risk asset, such as stocks. Each agent’s initial holdings and cash follow a uniform distribution, $S_{0}^{i}\sim U\left(0,Ns\right),C_{0}^{i}\sim U\left(0,Nc\right)$. To simulate the trading process accurately, we decompose every trading day into 49 sub-periods. When each cycle begins, a proportion of agents are randomly selected to enter the market, and each agent predicts the future value of the stock and then submits the order.

Assuming that all agents know the stock’s fundamental value of *P*^*f*^ and its historical price, *P*^*f*^ is given by:

$$p_{t+1}^{f}=p_{t}^{f}e^{\sigma_{f}}$$ (1)

*P*^*f*^ can be affected by good or bad news. To simulate this effect, we assume that there is a 1% possibility that *P*^*f*^ will rise (or decrease) sharply. We set the market impact range from 5% to 15%.

Investment strategy is assumed to consist of fundamental analysis, technical analysis and noise trading. In our model, each agent’s investment strategy is a combination of these three strategies. The return of the agent that is selected into the market in the interval (t,t + *τ*^*i*^) is predicted by:

$${\hat{r}}_{t,t+\tau^{i}}^{i}=\frac{1}{g_{1}^{i}+g_{2}^{i}+n^{i}}\left[g_{1}^{i}\frac{1}{\tau_{f}}\text{ln}\left(\frac{p_{t}^{f}}{p_{t}}\right)+g_{2}^{i}{\bar{r}}_{t}^{i}+n^{i}\varepsilon_{t}\right]+d\omega_{t}^{i}$$ (2)

$g_{1}^{i},g_{2}^{i},n^{i}$ represent the weights that are given to a fundamental analysis component, technical analysis component, and noise analysis component, respectively, and they obey standard uniform distributions. It is well known that the opinions of noise traders may change from optimistic to pessimistic and vice versa. We add a noise component ε_t_ that denotes the ‘noise’ factors that can make the opinion change. The noise component has zero mean and variance σ_ε_. $d\omega_{t}^{i}$ is used to simulate the overnight information disturbance, where d is a dummy at the opening of d = 1 in the trading period of d = 0. In the disturbing term of $\omega_{t}^{i}\sim N\left(0,\sigma_{open}\right)$,*τ*_*f*_ is the reference value of *τ* when the fundamentalist component for the mean reversion of the price to the fundamental value, and ${\bar{r}}_{t}^{i}$ is the moving average of historical return that is referred by an agent of technical analysis at time t. That is,

$${\bar{r}}_{t}^{i}=\frac{1}{\tau^{i}}\sum r_{t-j}$$ (3)

Each agent has a different time horizon of *τ*^*i*^. When the fundamental analysis component is given greater weight, the agents have a longer time horizon. That is, investments should be based on the long-term value of the stocks. When the technical analysis is given greater weight, the agents have a shorter time horizon and engage in short-term trading. Accordingly,

$$\tau^{i}=\left[\tau \frac{1+g_{1}^{i}}{1+g_{2}^{i}}\right]$$ (4)

The predicted price of agent i at time *t* + *τ*_*i*_ is given by:

$${\hat{p}}_{t+\tau^{i}}^{i}=p_{t}\text{exp}\left({\hat{r}}_{t,t+\tau^{i}}^{i}\tau^{i}\right)$$ (5)

When the agents obtain the stock’s predicted price, the decisions and transactions will be performed. The agents determine the declared price interval [*p*_*min*_, *p*_*max*_] according to the optimal bid-ask quote in the order book. TL is the degree of pre-trade transparency; for example, if TL = 5, *p*_*min*_ is the 5-bid price, and *p*_*max*_ is the 5-ask price. If the levels of instantaneous orders are less than TL, then the agent’s price interval is calculated as follows:

$$p_{min}=b-l\times TS$$ (6)

$$p_{max}=a+h\times TS$$ (7)

When *h*,*l*~*U*(0,*TL*), TS is the minimum quotation tick, *a* is the best ask price, and *b* is the best bid price. If *a* or *b* does not exist, that is, if there are no buy or sell orders, then the last transaction price should be taken as the current price. The agent must decide whether to place a buy (or sell) order after forming his or her expectation of future price. The size of the order should be determined by utility maximization. Assume that the agent is risk-averse and that each agent has a different risk aversion coefficient. An agent who focuses on fundamental analysis is more risk-averse than an agent who focuses on technical analysis, and an agent who focuses on fundamental analysis therefore has a greater risk aversion coefficient. Assume that this agent has the following CARA (constant absolute risk aversion) expected utility function:

$$\text{U}\left(W_{t}^{i},\alpha^{i}\right)=-e^{-\alpha^{i}W_{t}^{i}}$$ (8)

$$\alpha^{i}=\alpha \frac{1+g_{1}^{i}}{1+g_{2}^{i}}$$ (9)

where α is the reference level of risk aversion, and $W_{t}^{i}$ is the wealth of agent i at time t. The calculation method is

$$W_{t}^{i}=S_{t}^{i}p_{t}+C_{t}^{i}$$ (10)

$S_{t}^{i}\geq 0\;and\;C_{t}^{i}\geq 0$ hold the shares and cash amount, respectively, of agent i at time t. Assume that short selling and borrowing cash is restricted. Each agent can calculate the optimal volume of holding shares at time t according to the principle of maximizing utility, regardless of the friction in the market (i.e., trading fees, stamp duty):

$$\pi^{i}\left(p\right)=\frac{\text{ln}\left({\hat{p}}_{t,t+\tau^{i}}^{i}/\text{p}\right)}{\alpha^{i}V_{t}^{i}p}$$ (11)

where p is the declared price, and $V_{t}^{i}$ is the variance of returns that are expected by agent i. The calculation method is:

$$V_{t}^{i}=\frac{1}{\tau^{i}}\sum {\left[r_{t-j}-{\bar{r}}_{t}^{i}\right]}^{2}$$ (12)

Considering the existence of a price limits may cause a price to remain at the upper (lower) limits for a long time; thus, $V_{t}^{i}=0$. The agent’s demand for the stock will tend to infinity; therefore, we assume that the minimum value of $V_{t}^{i}$ is le-5.

If $\pi^{i}\left(p\right)>S_{t}^{i}$, the agent will submit a buy order, and the quantity is $\pi^{i}\left(p\right)-S_{t}^{i}$; if the agent cannot afford the number of stock, the number of buying is $C_{t}^{i}/p_{t}$.

If $\pi^{i}\left(p\right)<S_{t}^{i}$, the agent will submit a sell order, and the quantity is $S_{t}^{i}-\pi^{i}\left(p\right)$. Because short selling is restricted, the maximum sell volume is $S_{t}^{i}$.

If $\pi^{i}\left(p\right)=S_{t}^{i}$, the agent thinks that the existing portfolio is optimal, and the agent no longer submits orders.

Until now, the agent has completed a decision process and submitted an order that includes the agent’s ID, trading direction, declared price, declared volume, declared time, and withdrawal time.

### 2.2 Simulation design

In this paper, the trading mechanisms of an artificial stock market are designed to simulate China’s stock market as accurately as possible. According to the trading rules of the Shanghai stock exchange, each trading day is divided into 49 sub-periods. The first sub-period attempts to simulate the opening call auction process, and the opening price is calculated. The remaining 48 sub-periods represent the four-hour trading process of each trading day. Considering that the trading volume during the opening period is generally greater than that during other trading periods of the day and the order book will be cleared after the market closed at the end of each trading day, it is necessary to generate enough orders to form a new order book at the beginning of each trading day. Therefore, we assume that the agent enters the market with a higher probability λ_open_ and keep the same probability λ in the other sub-period.

Before conducting our simulations, appropriate parameters were set and calibrated to generate a time series to show several stylized facts of real stock markets. The control parameters of the simulations are shown in Table 1. At the beginning of each simulation, the value of the fundamental price is 10. No agent was allowed to hold more than 100 shares of stock and 1,000 units in cash. The tick size and the range of price limits in this calibration model were the same as the tick size and the range of price limits in the Shanghai and Shenzhen A-shares market.

**Table 1:** Parameters of the artificial stock market.

Parameters	Value	Description
**N**	**1,000**	**Number of agent**
***P***_***f***_**(0)**	**10**	**Initial value of stock fundamental value**
***σ***_***f***_	**1E-3**	**Daily volatility of stock fundamental value**
***σ***_***e***_	**1E-4**	**Standard deviation of the composition of noise**
***σ***_***open***_	**1E-3**	**Standard deviation of the disturbance of overnight information**
**τ**	**40**	**Reference value of agent in investment period**
**α**	**0.1**	**Reference value of risk aversion**
**Ns**	**100**	**Maximum initial stock holdings**
**Nc**	**1,000**	**Maximum initial cash holdings**
**TS**	**0.01**	**Tick size**
**TL**	**5**	**Market reveals gear in order books**
***λ***_***open***_	**0.08**	**Proportion of agents entering market at opening**
**λ**	**0.02**	**Proportion of agents entering market**
**PL**	**0.1**	**Range of price limits**

### 2.3 Calibration of the artificial stock market

Many trends in the stock market have been revealed. Stanley’s group has demonstrated that the tail distributions of stock returns in the US market obey an inverse cubic law [32–34]. In the Chinese stock market, studies have indicated that the distributions of returns at different microscopic timescales exhibit power-law tails [35] and can be fitted by a q-Gaussian distribution [36]. In this section, we calibrate the artificial stock market with the actual transaction data to test the frequency hitting price limits, fat tails and volatility clustering that are found in the actual stock market.

The initial price of the stock in the artificial stock market is set to 10, and the simulated price varies from 5 to 20. Therefore, we selected three similar stocks: China Television Media (CTM), Dongfeng Electronic Technology (DETC) and Shanghai Potevio (SP). We use 2000 five-minute trading data since the first trading day in 2011. These stocks hit price limits with higher frequency, and we compare them with the simulated data. The results are shown in Table 2.

**Table 2:** Comparison of statistical properties.

Sample	Number of hitting	Mean	Standard deviation	Skewness	Kurtosis	JB statistics
**CTM**	**14**	**-8.51E-06**	**0.003**	**1.0702**	**18.6769**	**2.09E+04**
**DETC**	**17**	**3.57E-05**	**0.0036**	**0.6606**	**8.0378**	**2.26E+03**
**SP**	**26**	**3.88E-05**	**0.0043**	**2.818**	**56.0748**	**2.37E+05**
**CDA**	**14**	**2.71E-06**	**0.0031**	**1.1419**	**39.8214**	**1.13E+05**

Table 2 shows that the return series’ statistical properties of the three stocks and the simulated data (CDA) are very similar. For each stock, the kurtosis is significantly greater than that found in Gaussian distributions with a kurtosis of 3; this result indicates that tails are decaying extremely slowly. This finding is consistent with prior empirical results for Chinese stocks [35, 36]. All four return series have the stylized facts of kurtosis and fat tails.

**Fig 1:**
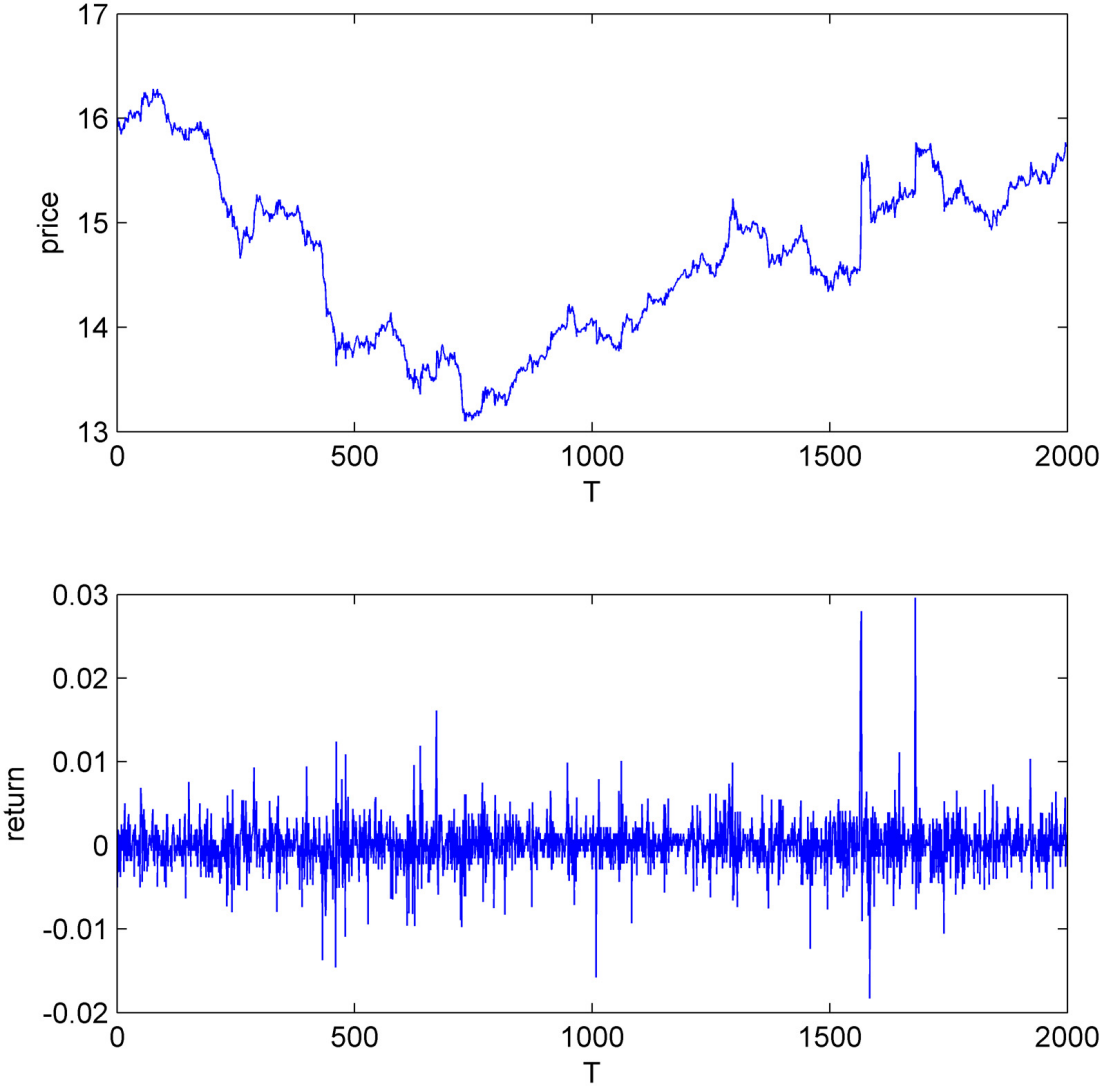
The price and return series of China Television Media.

**Fig 2:**
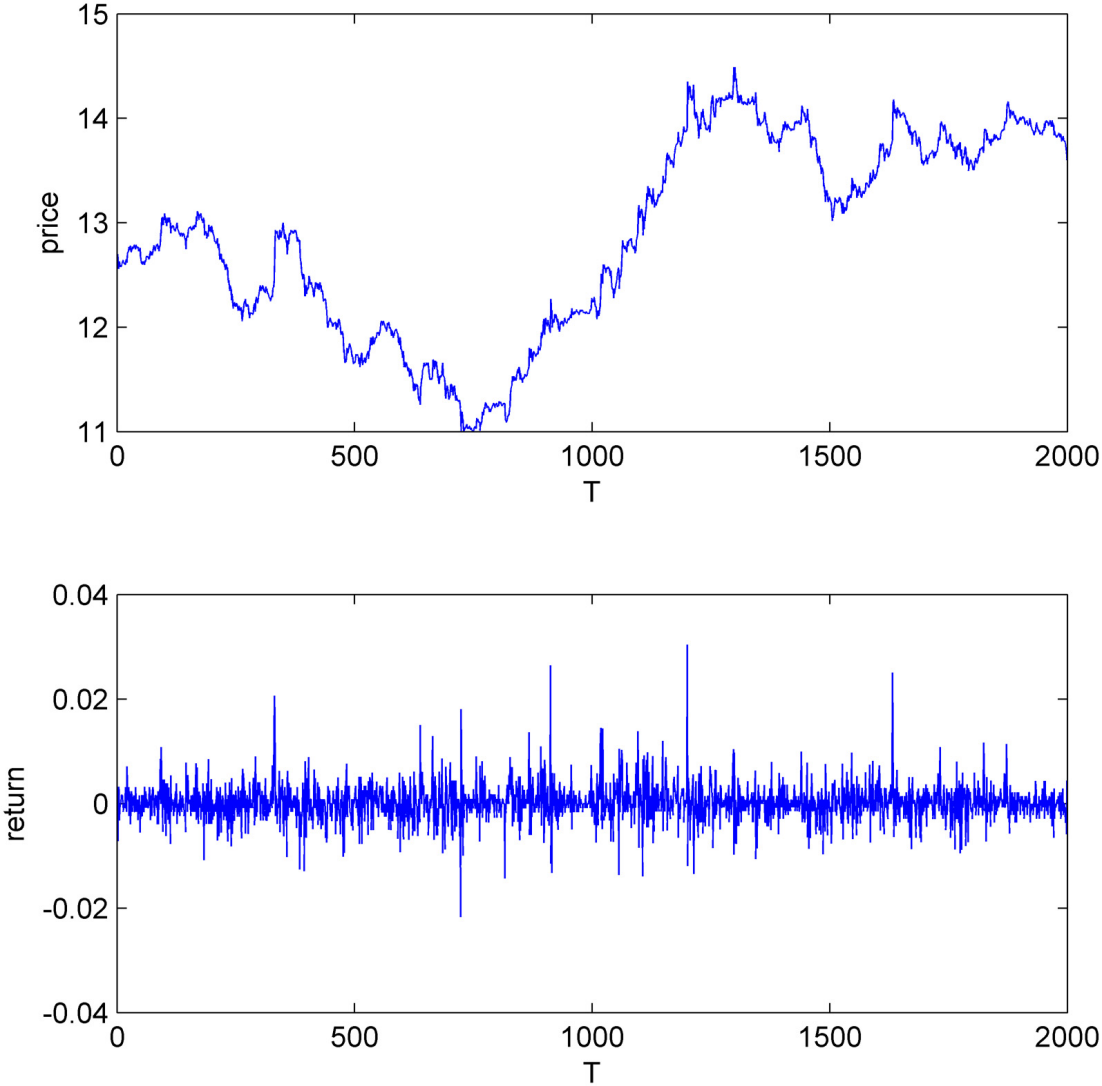
The price and return series of Dongfeng Electronic Technology.

**Fig 3:**
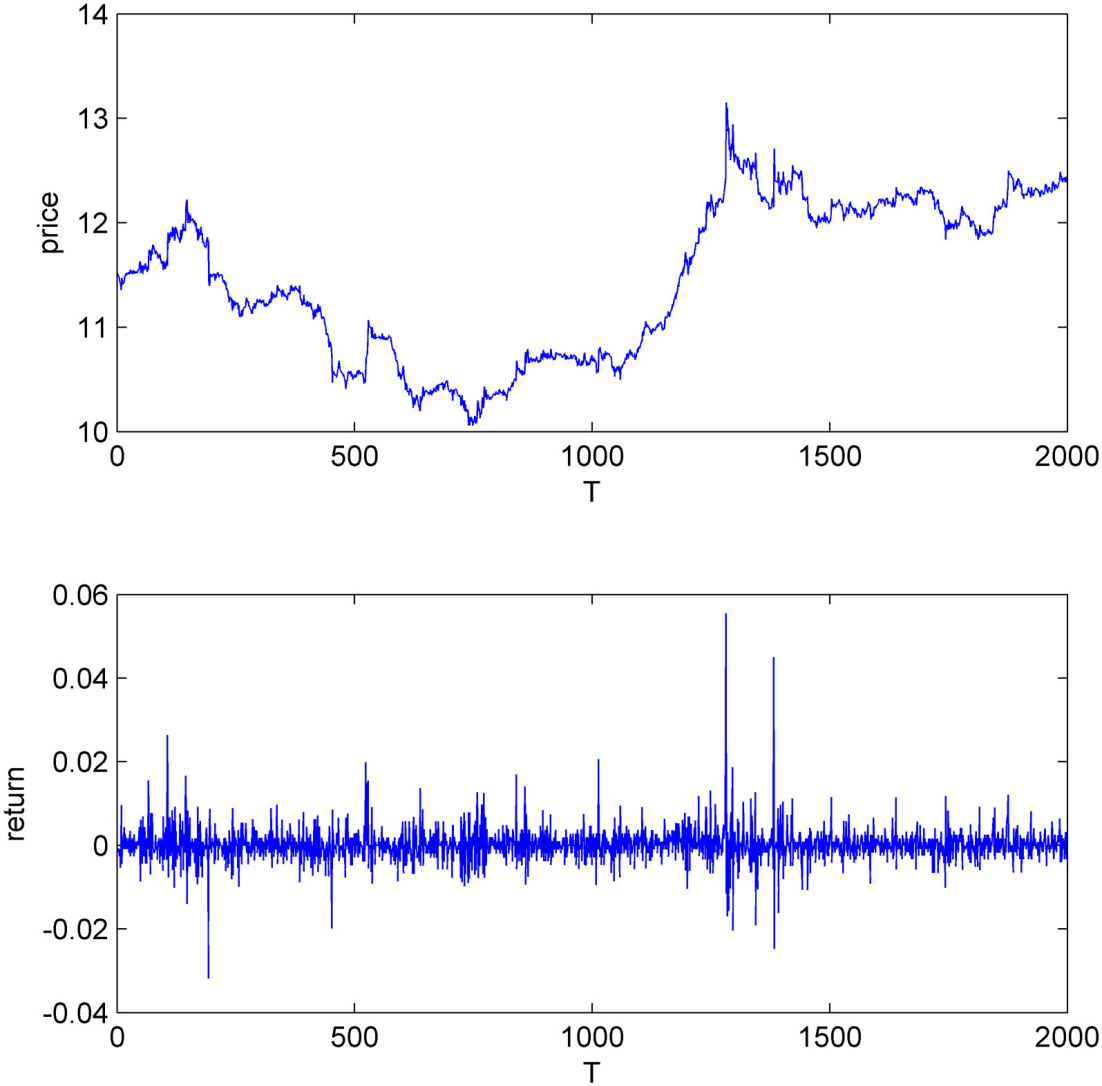
The price and return series of Shanghai Potevio.

**Fig 4:**
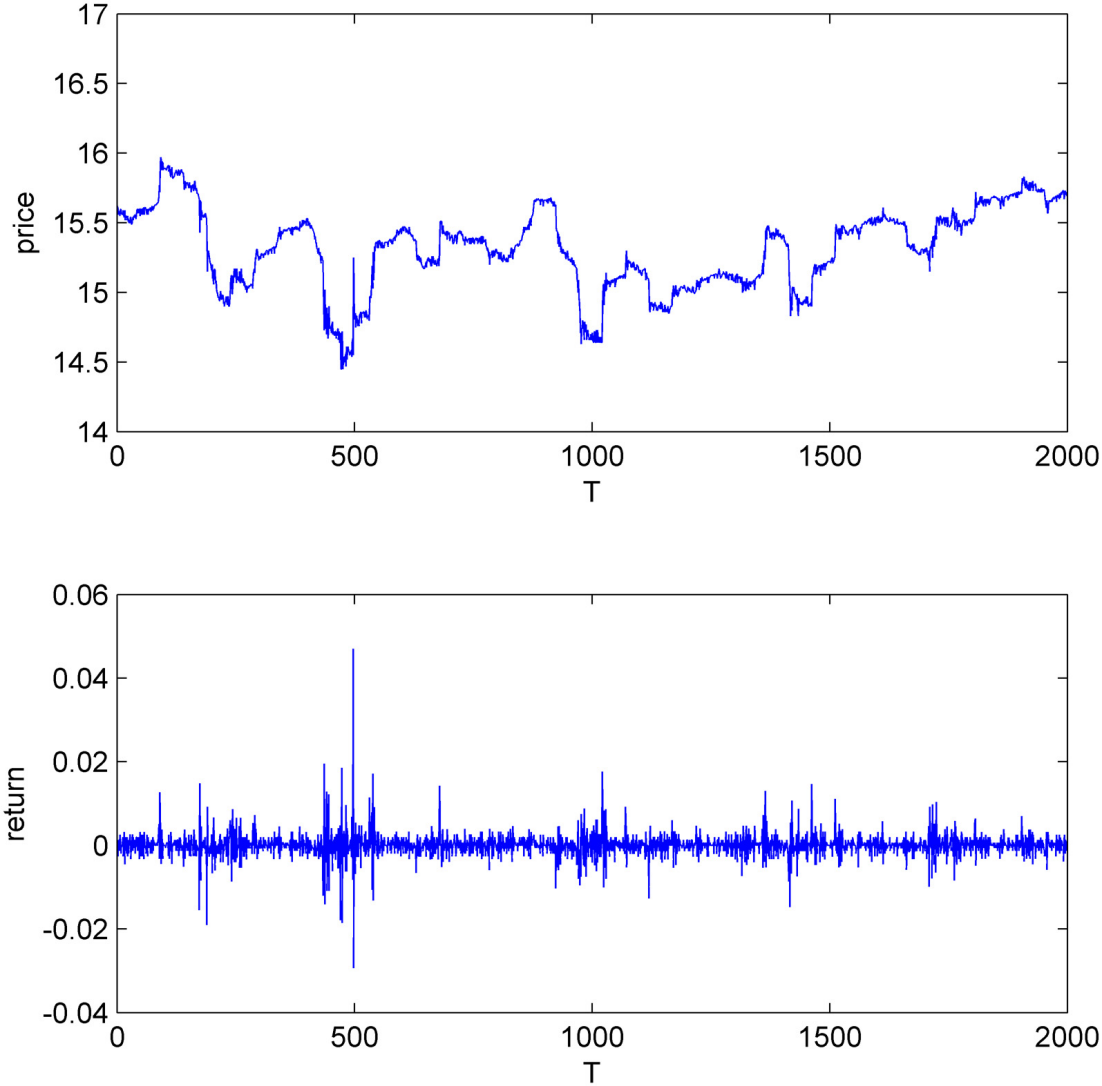
The price and return series of CDA.

Figs 1–4 display the price and return series of the three stocks and the simulated data. We found that the five-minute yields vary between -2% and 2%. The price volatility of the three stocks and CDA have similar trends. We also find that all the four return series have the same first-order autocorrelation and the autocorrelation coefficients range from -0.2 to 0 (Figures A-H in S1 File).

Figs 5–8 show distribution fitting, and Figs 9–12 show QQ graphs of the return series. The two diagrams show that all four samples are characterized as fat tails. This finding means that our artificial stock market can simulate the essential features of the real stock market.

**Fig 5:**
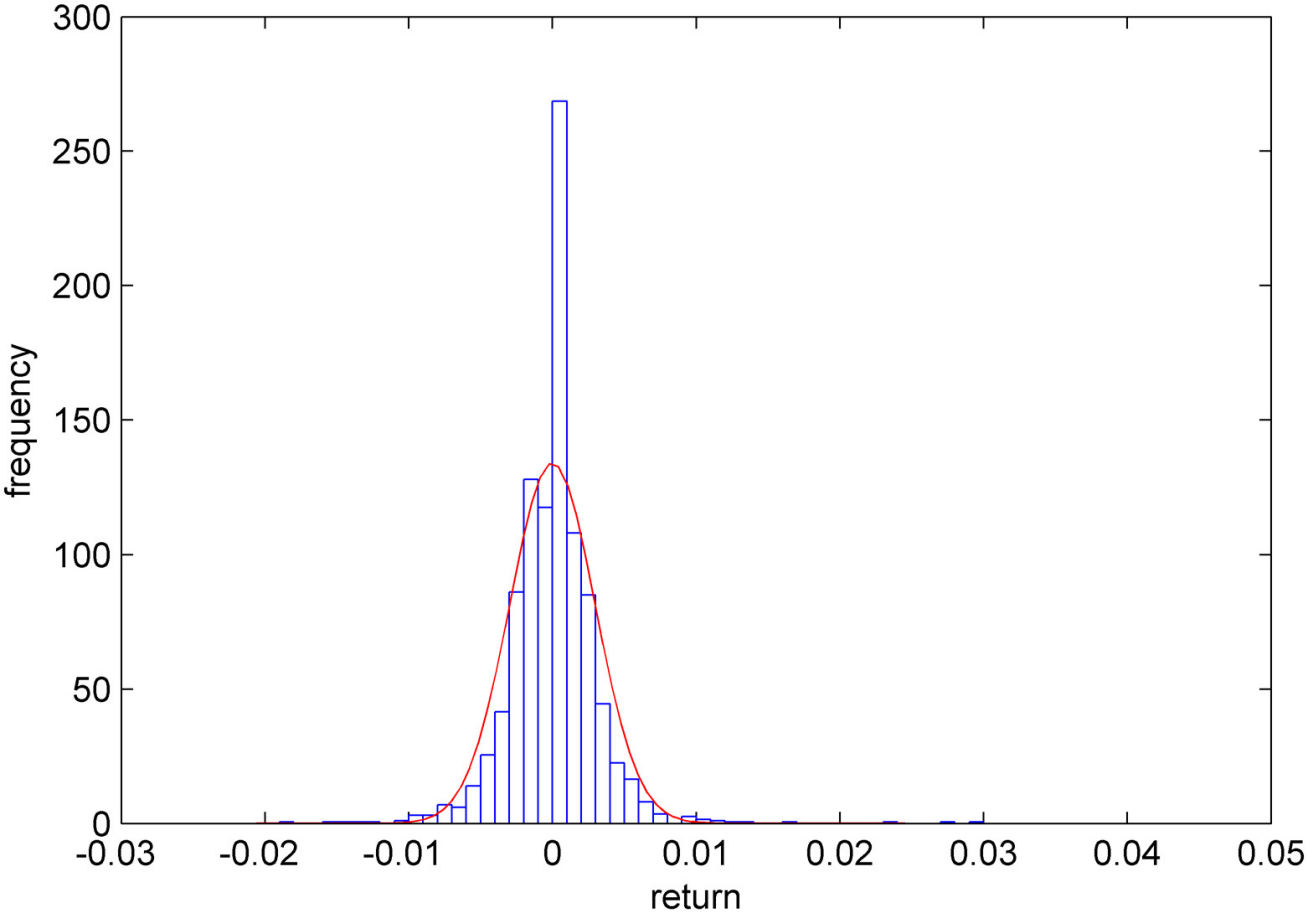
The yield distribution fitting diagram of China Television Media.

**Fig 6:**
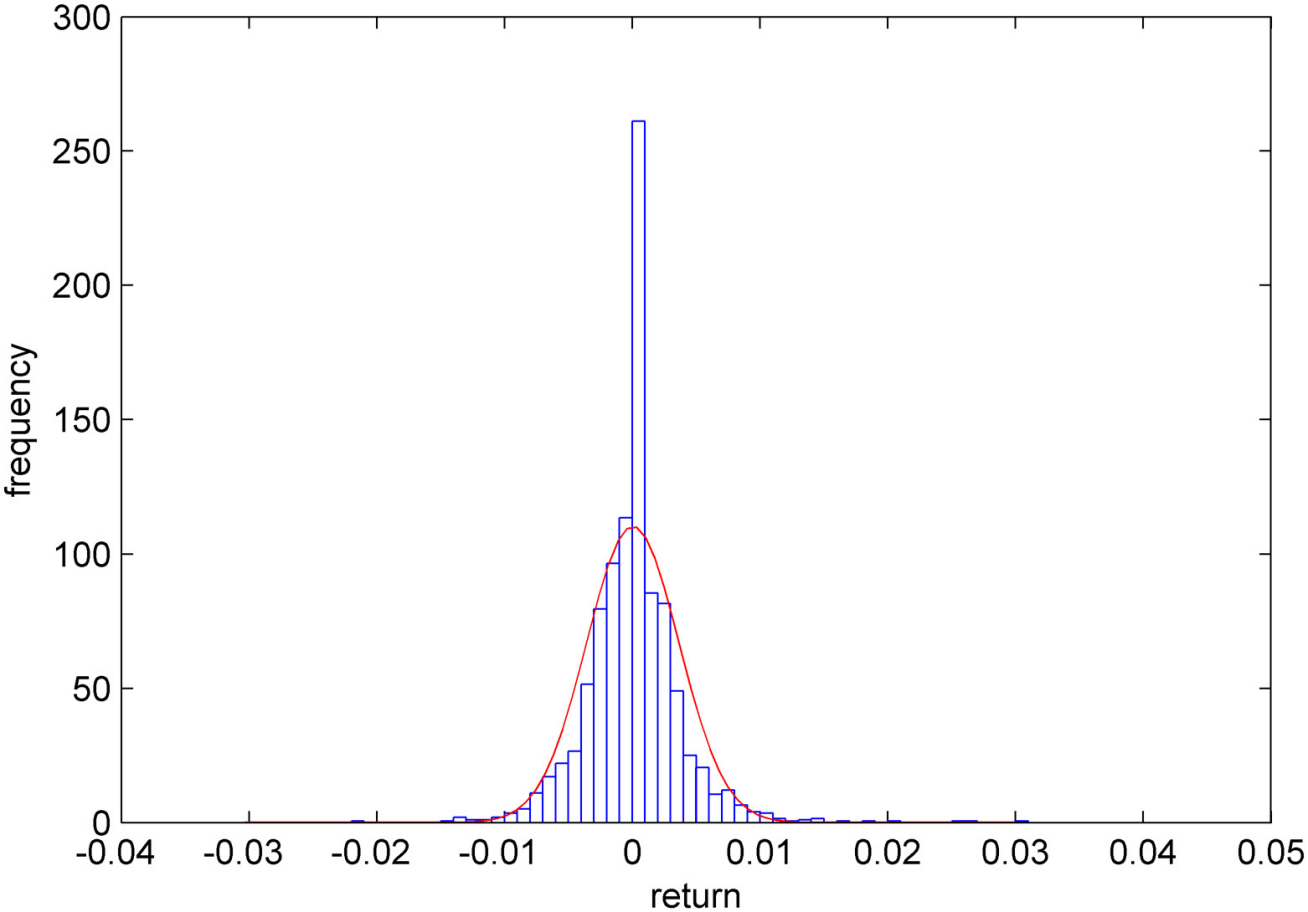
The yield distribution fitting diagram of Dongfeng Electronic Technology.

**Fig 7:**
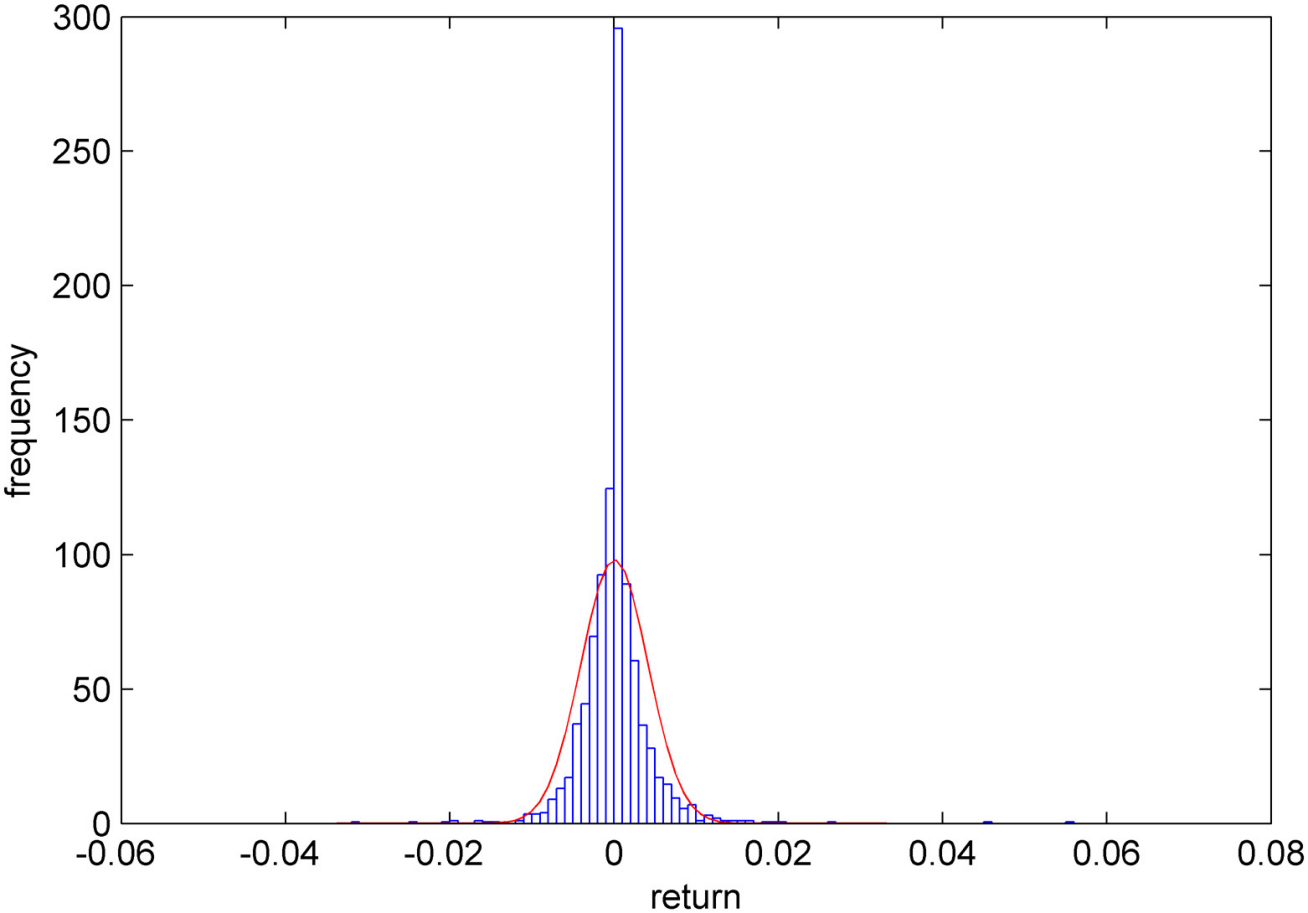
The yield distribution fitting diagram of Shanghai Potevio.

**Fig 8:**
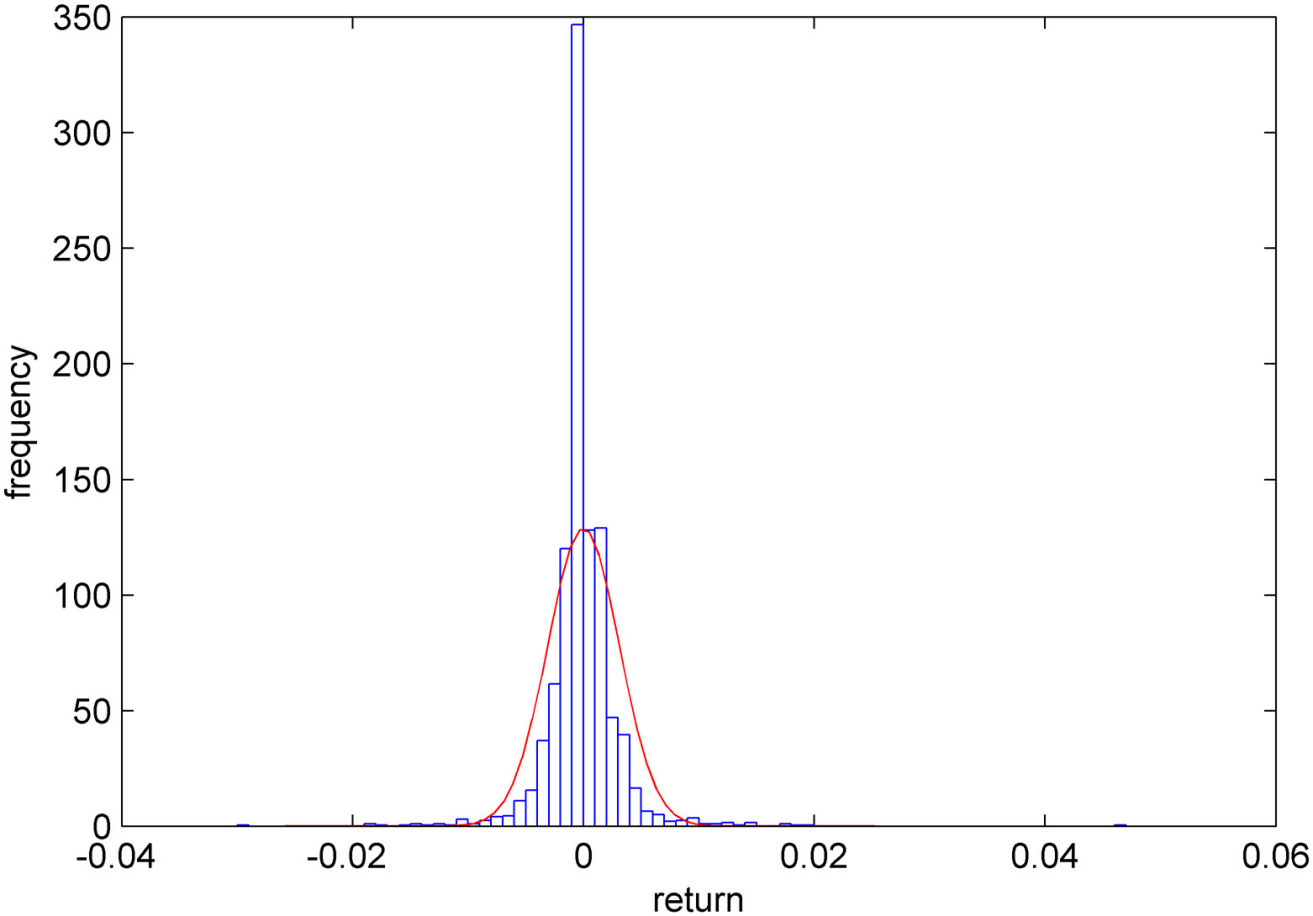
The yield distribution fitting diagram of CDA.

**Fig 9:**
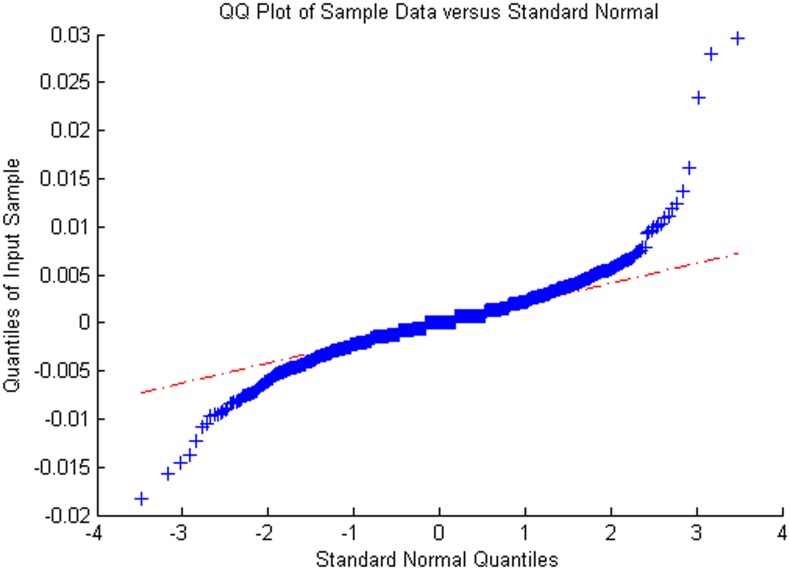
The return series QQ graph of China Television Media.

**Fig 10:**
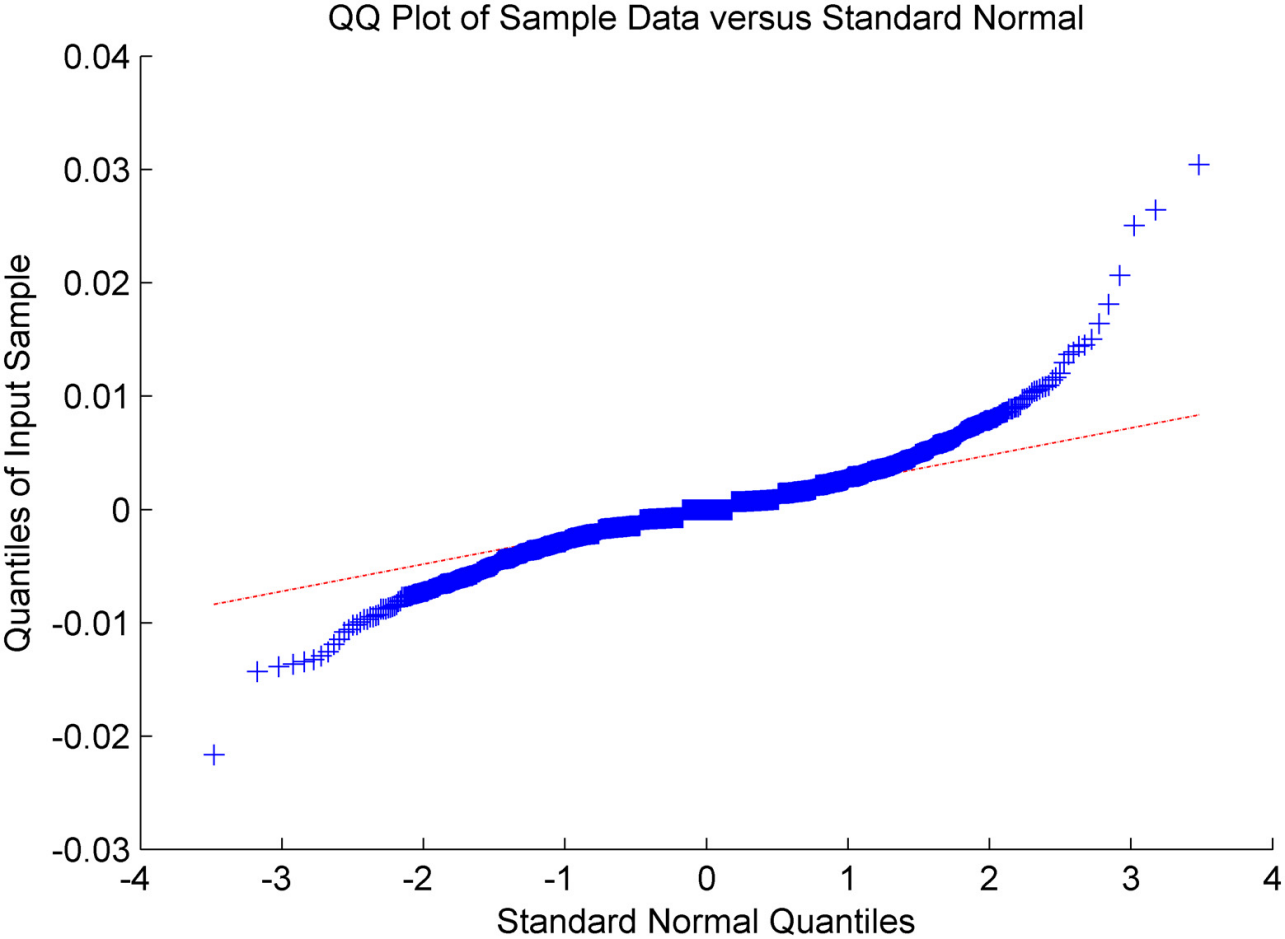
The return series QQ graph of Dongfeng Electronic Technology.

**Fig 11:**
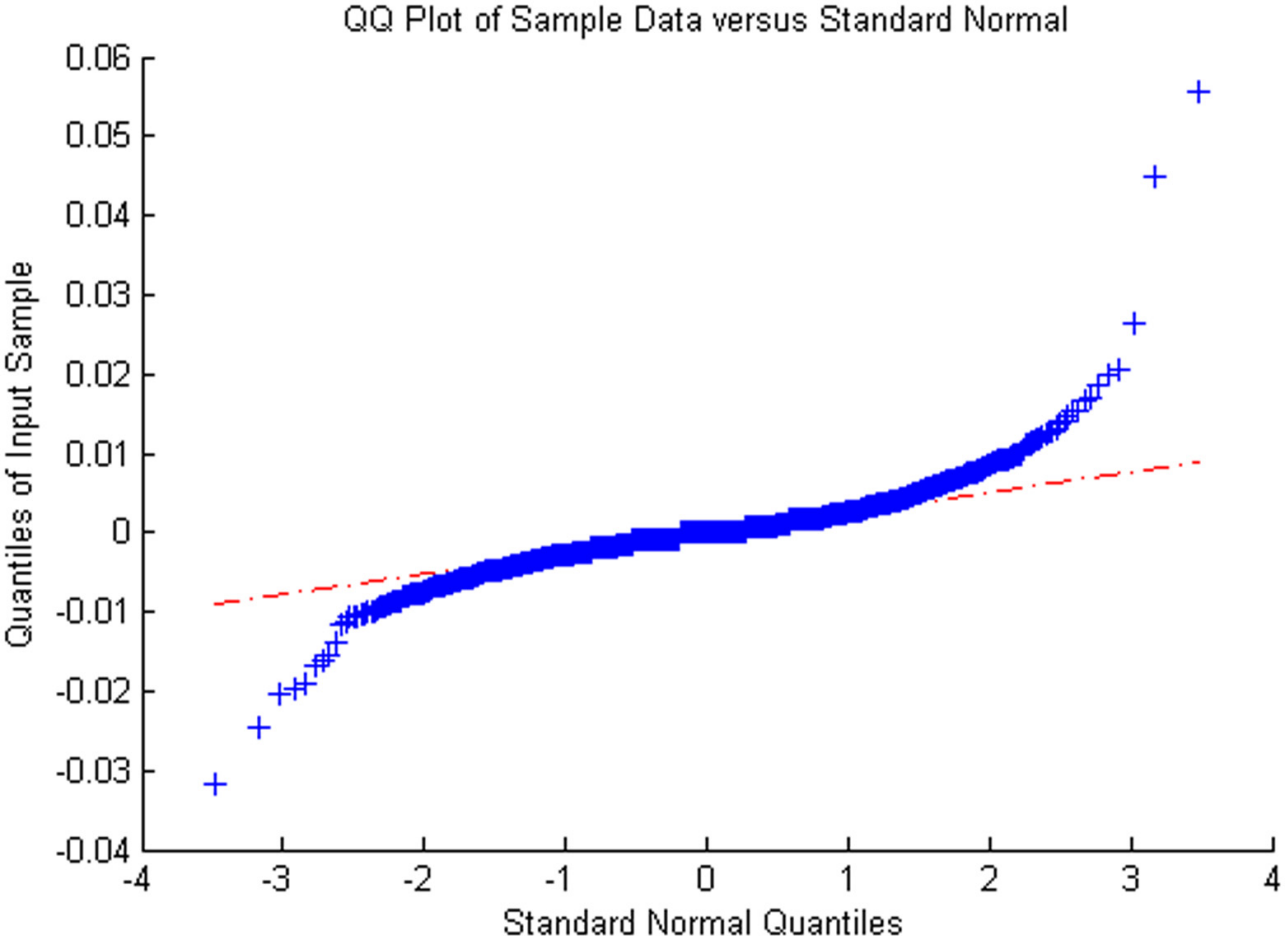
The return series QQ graph of Shanghai Potevio.

**Fig 12:**
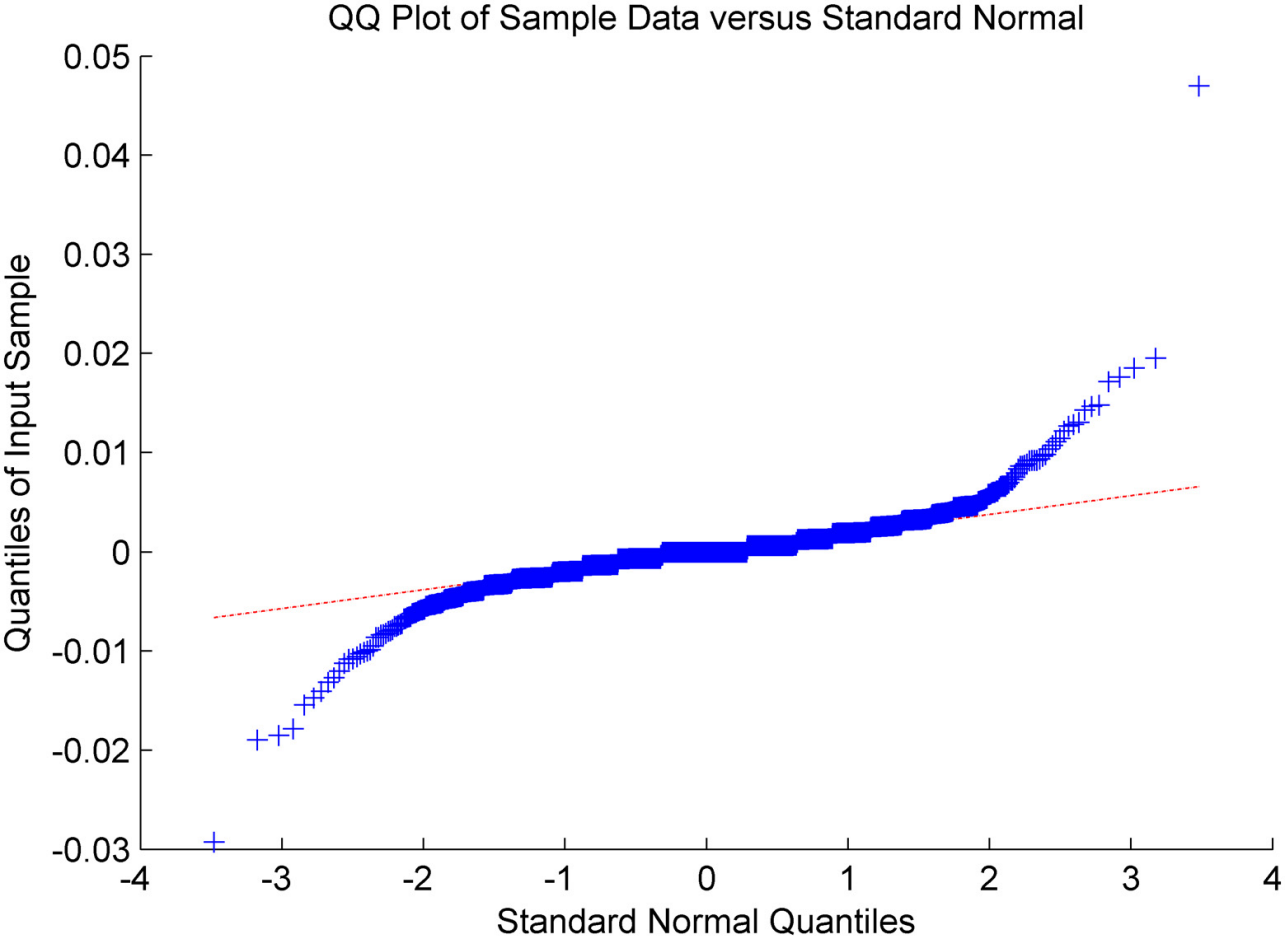
The return series QQ graph of CDA.

To make the conclusion more convincing, we performed a volatility clustering test with GARCH(1,1); for details, see Table 3. The conditional variance equation of GARCH(1,1) is as follows:

$$\sigma_{t}^{2}=\omega +\alpha u_{t-1}^{2}+\beta \sigma_{t-1}^{2}$$ (13)

**Table 3:** Coefficient of the GARCH Model.

	α	β	α + β	Log likelihood
**CTM**	**0.4686**	**0.2191**	**0.6877**	**8456.18**
**DETC**	**0.5992**	**0.1693**	**0.7685**	**8912.04**
**SP**	**0.4997**	**0.4419**	**0.9416**	**8237.44**
**CDA**	**0.4661**	**0.4424**	**0.9085**	**9194.43**

If α and β are significantly greater than zero, then when the sum of α and β is greater, the lasting impact is longer and the volatility clustering is more obvious. From the results of Table 3, the four samples’ α and *β* values are significantly positive, and the value of *α* + *β* from in the artificial stock market is greater than 0.9, which accords with the actual stock market. Therefore, the volatility clustering feature is significant.

After this comparison, the simulated data have many similarities with the actual stock market concerning its statistical characteristics. The artificial stock market can be used to simulate high frequency price limits hitting behavior in the Shanghai Securities exchange. The artificial stock market can also be used to research the inter-day effects of price limits.

## 3 Simulation Results

The simulation experiments were conducted with different settings; one group is without price limits, and the other group has price limits that were set at a 10% level, which is the same as China’s A-share market (regardless of the ST and * ST shares). We performed 30 experiments in each group by setting different initial seeds (the initial seeds were 1,000, 2,000, 3,000, … 30,000). Each experiment simulated 400 trading days and 19,600 transaction sub-periods. We chose a 15-day window from Day-7 to Day+7 to test the inter-day effects of price limits. Day+0 was the event day, namely, the day that the price limits was hit. Day+1 to Day+7 represent the 1st to the 7th trading days after the event day, respectively, and Day-7 to Day-1 represent the 7th to the 1st trading days before the event day, respectively. When the stock price increases, the price of the upper limits hitting stocks must meet the following conditions:

$$H_{t}+0.005\geq 1.1\times C_{t-1}$$ (14)

When the stock price decreases, the price of the lower limits hitting stocks must meet the following conditions:

$$L_{t}-0.005\leq 0.9\times C_{t-1}$$ (15)

In these equations, *H*_*t*_ and *L*_*t*_ denote the highest and lowest prices of the stocks, respectively, and *C*_*t*_ represents the closing price.

### 3.1 Test for the volatility spillover effect

If there is a volatility spillover effect, stocks will have higher volatility after the price limits hitting day. The volatility is measured by the square of return, namely, *V*_*i*,*j*_ = (*r*_*i*,*j*_)^2^, where *r*_*i*,*j*_ = *ln*(*C*_*t*_/*C*_*t*−1_) is the daily return of stock i at time t, and *C*_*t*_ is the closing price. We compared the volatility of the two groups after the price-limits-hitting day to investigate whether price limits can lead to a volatility spillover effect.

The mean and median of volatility over the 15-day window of the two groups are reported in Table 4. When the stock price rises, the volatility on Day+1 of the group with price limits is significantly higher than before the event day, and then the volatility returns to normal levels on subsequent trading days. However, the volatility of the group without price limits already returns to normal levels on Day+1, and this group has less volatility than the group with price limits at a 1% significance level. This result provides evidence to support the volatility spillover effect of price limits. For the downward price movement, the volatility of the group with price limits on Day+1 to Day+3 is higher than normal levels, and then the volatility returns to normal levels on subsequent trading days. Although the volatility of the group without price limits returns to normal levels on Day+2, the volatility of the group with price limits on Day+1 is greater than the volatility of the group without price limits at a 1% significance level. This result also supports a volatility spillover effect. Therefore, price limits significantly increase the volatility of the stock market.

**Table 4:** The simulation results for the volatility spillover effect.

Day	with price limits			without price limits	
**upward price movement**					
**-7**	**0.602**	**(0.104)**		**1.177**	**(0.092)**
**-6**	**0.706**	**(0.070)**		**0.916**	**(0.060)**
**-5**	**0.413**	**(0.072)**		**0.671**	**(0.111)**
**-4**	**0.400**	**(0.107)**		**0.539**	**(0.084)**
**-3**	**0.402**	**(0.048)**		**0.415**	**(0.063)**
**-2**	**0.830**	**(0.113)**		**1.455**	**(0.159)**
**-1**	**0.897**	**(0.082)**		**1.532**	**(0.116)**
**0**	**9.018**	**(9.102)**	**<<**	**14.618**	**(13.903)**
**1**	**1.363**	**(0.948)**	**>>**	**0.438**	**(0.119)**
**2**	**0.446**	**(0.081)**		**0.979**	**(0.144)**
**3**	**0.254**	**(0.103)**		**0.337**	**(0.103)**
**4**	**0.401**	**(0.078)**		**0.567**	**(0.103)**
**5**	**0.491**	**(0.085)**		**1.033**	**(0.099)**
**6**	**0.637**	**(0.052)**		**0.607**	**(0.075)**
**7**	**0.393**	**(0.105)**		**0.378**	**(0.104)**
**downward price movement**					
**-7**	**0.275**	**(0.069)**		**0.423**	**(0.065)**
**-6**	**0.999**	**(0.105)**		**1.219**	**(0.087)**
**-5**	**0.715**	**(0.072)**		**1.160**	**(0.095)**
**-4**	**0.528**	**(0.063)**		**1.023**	**(0.061)**
**-3**	**0.353**	**(0.090)**		**0.335**	**(0.072)**
**-2**	**0.255**	**(0.062)**		**0.570**	**(0.081)**
**-1**	**1.272**	**(0.086)**		**0.803**	**(0.071)**
**0**	**10.917**	**(11.080)**	**<<**	**20.507**	**(18.593)**
**1**	**3.085**	**(1.731)**	**>>**	**2.725**	**(0.493)**
**2**	**1.146**	**(0.463)**		**1.275**	**(0.299)**
**3**	**0.833**	**(0.268)**		**0.424**	**(0.110)**
**4**	**0.560**	**(0.141)**		**0.837**	**(0.124)**
**5**	**0.531**	**(0.109)**		**0.616**	**(0.055)**
**6**	**0.988**	**(0.112)**		**1.167**	**(0.118)**
**7**	**0.708**	**(0.132)**		**0.876**	**(0.093)**

Note: Volatility is measured as follows: *V*_*i*,*j*_ = (*r*_*i*,*j*_)^2^. The values that are shown in this table are multiplied by 1,000; the volatility of each trading day is taken from the average value (median values are shown in brackets). “>>” (“<<”) means that the medians on the left (right) are greater than the medians on the right (left) at a 1% significance level according to Wilcoxon’s rank-sum test.

### 3.2 Test for the trading interference effect

If price limits prevent trading, stocks become less liquid, which may cause intensified trading activity on subsequent days. We expect to find that trading volume increases for the stocks that are grouped on the day after a price limits hitting day, which indicates continued intense trading. The implication is that price limits prevent rational trading on the event day and indicates a harmful interference to liquidity. The liquidity of stocks is measured by a logarithmic percentage change in stock trading volume, namely, T*C*_*t*,*j*_ = ln(*TVOL*_*t*,*j*_/*TVL*_*t*−1,*j*_) × 100, and *TVOL*_*t*,*j*_ is the trading volume of j stock on Day t. The results of this test are shown in Table 5.

**Table 5:** The simulation results for the trading interference effect.

Day	_with price limits_			without price limits	
**upward price movement**					
**-7**	**2.74%**	**(-0.81%)**		**4.31%**	**(0.73%)**
**-6**	**-0.63%**	**(-3.92%)**		**-3.38%**	**(-3.79%)**
**-5**	**5.55%**	**(0.36%)**		**4.69%**	**(1.02%)**
**-4**	**-3.45%**	**(-2.79%)**		**-2.20%**	**(-2.71%)**
**-3**	**-2.34%**	**(2.89%)**		**-4.27%**	**(3.41%)**
**-2**	**-7.31%**	**(-2.15%)**		**-2.25%**	**(1.93%)**
**-1**	**7.39%**	**(-0.78%)**		**5.07%**	**(-3.07%)**
**0**	**-133.80%**	**(-147.79%)**	**<<**	**-4.86%**	**(-2.84%)**
**1**	**134.08%**	**(138.72%)**	**>>**	**8.95%**	**(5.00%)**
**2**	**0.10%**	**(0.62%)**		**-2.44%**	**(-3.27%)**
**3**	**2.17%**	**(-1.65%)**		**-0.40%**	**(-1.55%)**
**4**	**-4.79%**	**(-4.16%)**		**-3.45%**	**(-5.90%)**
**5**	**-2.22%**	**(-0.76%)**		**1.24%**	**(-0.86%)**
**6**	**-6.00%**	**(-2.81%)**		**-2.59%**	**(-3.03%)**
**7**	**9.76%**	**(8.35%)**		**2.28%**	**(1.15%)**
**downward price movement**					
**-7**	**-4.52%**	**(0.78%)**		**-5.28%**	**(-2.49%)**
**-6**	**-7.86%**	**(-3.67%)**		**2.57%**	**(-2.90%)**
**-5**	**7.45%**	**(1.48%)**		**2.02%**	**(2.27%)**
**-4**	**5.55%**	**(-3.62%)**		**-1.47%**	**(-1.91%)**
**-3**	**2.21%**	**(-0.13%)**		**0.52%**	**(-1.02%)**
**-2**	**-4.95%**	**(-4.00%)**		**-1.66%**	**(-2.84%)**
**-1**	**-26.12%**	**(-1.33%)**		**-2.02%**	**(1.90%)**
**0**	**-94.11%**	**(-49.49%)**	**<<**	**3.62%**	**(10.02%)**
**1**	**140.88%**	**(70.49%)**	**>>**	**1.86%**	**(1.75%)**
**2**	**-2.76%**	**(-9.71%)**		**2.48%**	**(2.28%)**
**3**	**1.57%**	**(0.84%)**		**1.53%**	**(-0.17%)**
**4**	**-7.92%**	**(-5.32%)**		**-5.85%**	**(-7.34%)**
**5**	**-1.94%**	**(-3.28%)**		**-2.22%**	**(-6.06%)**
**6**	**-0.04%**	**(1.10%)**		**-1.32%**	**(-0.83%)**
**7**	**-1.42%**	**(-2.10%)**		**-1.43%**	**(0.36%)**

Note: The trading volumes of each trading day are taken from the average value (the median values are shown in brackets). “>>” (“<<”) means that the medians on the left (right) are greater than the medians on the right (left) at a 0.01 significance level according to Wilcoxon’s rank-sum test.

From the results that are shown in Table 5, when price rises, the price limits trading volume of the groups with price limits decreases on Day0, and the falling range of this trading volume is -133.80%. On the next day (Day1), the trading volumes rise significantly, and the rising range is 134.08%. This phenomenon is also found when prices fall. However, in the group without price limits, there is no obvious change in trading volume on the event day and the next day. Our findings support the existence of a trading interference effect and are consistent with the results obtained by Kim and Rhee [4].

### 3.3 Test for the delayed price discovery effect

The delayed price discovery effect claims that positive (negative) overnight returns may occur after a price limits is hit. To test the delayed price discovery effect, we calculate the close-to-open return, $r\left(O_{0}C_{1}\right)=ln\left(\frac{C_{0}}{O_{0}}\right)$, on the event day and the open-to-close returns, *r*(*C*_0_*O*_1_) = *ln*(*O*_1_/*C*_0_), after the event day. *O*_0_ and *C*_0_ denote the opening and closing price on the event day, respectively, and *O*_1_ denotes the opening price on Day1. If the return is positive, it is marked as (+); if the return is negative, it is marked as (-). If the return is 0, it is marked as (0). Therefore, there can be the following nine types of possible combinations for the two returns above: [+ +], [0, +], [+, -], [0,–], [-, +], [–, 0], [–,–], [+, 0], and [0, 0]. For upper limits hitting, regard [+ +] and [0, +] as price continuations, [+, -], [0,–], [-, +], [–, 0] and [–,–] as price reversals, and [+,0] and [0,0] as price unchanged. For lower limits hitting, regard [–,–] and [0,–] as price continuations, [+,+], [0,+], [+,0], [+,-], and [-,+] as price reversals, and [–,0] and [0,0] as price unchanged. Table 6 shows the probability of price continuations, price reversals and no changes.

**Table 6:** The simulation results for the delayed price discovery effect.

price behavior	upward price movement		downward price movement	
	with price limits	without price limits	with price limits	without price limits
**continuations**	**0.50**	**0.43**	**0.41**	**0.41**
**reversal**	**0.50**	**0.57**	**0.38**	**0.46**
**unchanged**	**0**	**0**	**0.21**	**0.13**

The delayed price discovery effect means that the existence of price limits will hinder the process of stock price discovery, and the group with price limits will have a significant likelihood of price continuations. According to Table 6, when the price rises, the frequency of price continuations in the group with price limits is higher than in the group without price limits, which is 0.5 and 0.43, respectively. Therefore, we observe evidence of a delayed price discovery with upward price movement. However, when the stock price falls, the probability of the two groups are equal at 0.41. Thus, when upper price limits are imposed, the value of price continuations will increase. This finding supports the existence of a delayed price discovery effect for upper, not lower, price limits.

However, when stock prices rise, the probability of price continuations in the group with price limits is not much higher than this probability in the group without price limits. The evidence of delayed price discovery is not powerful. At this point, we introduce a new variable, *Dev*_*t*_, to measure the speed of price discovery. This variable is the absolute value of the difference between stock price and fundamental value, namely, $Dev_{t}=\left|p_{t}-p_{t}^{f}\right|$. If the delayed price discovery effect exists, the deviation value will increase significantly.

Table 7 shows the results. In the group with price limits, it is obvious that when stock prices rise, the deviation value on Day 1 is significantly larger than normal levels, whereas in the group without price limits, the deviation value is rather steady. This phenomenon supports the price discovery effect. However, when stock prices fall, the deviation values of both groups have the same change trend. This finding shows that lower price limits cannot cause a delayed price discovery effect. This result is consistent with the outcome of the price continue probability’s test.

**Table 7:** Deviation value test for the delayed price discovery effect.

Day	_with price limits_			_without price limits_	
**upward price movement**					
**-7**	**0.083**	**(0.050)**		**0.095**	**(0.047)**
**-6**	**0.081**	**(0.050)**		**0.087**	**(0.054)**
**-5**	**0.081**	**(0.048)**		**0.103**	**(0.058)**
**-4**	**0.080**	**(0.045)**		**0.080**	**(0.043)**
**-3**	**0.091**	**(0.053)**		**0.088**	**(0.054)**
**-2**	**0.082**	**(0.044)**		**0.072**	**(0.044)**
**-1**	**0.093**	**(0.056)**		**0.164**	**(0.069)**
**0**	**0.110**	**(0.042)**		**0.281**	**(0.054)**
**1**	**0.340**	**(0.368)**	**>>**	**0.133**	**(0.072)**
**2**	**0.058**	**(0.044)**		**0.120**	**(0.058)**
**3**	**0.073**	**(0.050)**		**0.115**	**(0.077)**
**4**	**0.113**	**(0.083)**		**0.120**	**(0.071)**
**5**	**0.093**	**(0.066)**		**0.097**	**(0.054)**
**6**	**0.080**	**(0.057)**		**0.095**	**(0.063)**
**7**	**0.108**	**(0.072)**		**0.114**	**(0.074)**
**downward price movement**					
**-7**	**0.066**	**(0.052)**		**0.088**	**(0.054)**
**-6**	**0.068**	**(0.043)**		**0.105**	**(0.049)**
**-5**	**0.096**	**(0.060)**		**0.096**	**(0.057)**
**-4**	**0.091**	**(0.059)**		**0.095**	**(0.061)**
**-3**	**0.084**	**(0.055)**		**0.100**	**(0.062)**
**-2**	**0.064**	**(0.051)**		**0.110**	**(0.051)**
**-1**	**0.087**	**(0.042)**		**0.080**	**(0.042)**
**0**	**0.101**	**(0.052)**		**0.093**	**(0.065)**
**1**	**0.241**	**(0.194)**		**0.252**	**(0.151)**
**2**	**0.265**	**(0.247)**		**0.557**	**(0.272)**
**3**	**0.159**	**(0.109)**		**0.125**	**(0.070)**
**4**	**0.076**	**(0.058)**		**0.079**	**(0.060)**
**5**	**0.081**	**(0.052)**		**0.078**	**(0.060)**
**6**	**0.071**	**(0.044)**		**0.077**	**(0.050)**
**7**	**0.070**	**(0.039)**		**0.059**	**(0.045)**

Note: The *Dev*_*t*_ value of each trading day is taken from the average value (the median value is in brackets). “>>” means that the medians on the left are significantly greater than the medians on the right at a 1% significance level according to Wilcoxon’s rank-sum test.

## 4. Conclusion

In this paper, we explored the inter-day effects of price limits on China’s stock market. Because the trading data in the real stock market confuses some financial factors, it is difficult to isolate the impact of price limits from other factors. Therefore, we built an artificial order-driven stock market by referring to Chiarella et al. [25], Chiarella and Iori [29] and Li et al. [30]. We modified the artificial stock market considering the unique trading mechanisms and regulatory conditions of China’s stock market. Then, we conducted a series of simulations with and without price limits.

We found that price limits can increase volatility after the price limits are hit. This phenomenon indicates that a volatility spillover effect exists. The main reason for this effect may be investor overreaction. Our findings also support the trading interference effect because trading volume increases after the price limits hitting day. If upper price limits are imposed on the stock market, the price discovery will be delayed, whereas this phenomenon is not apparent when lower price limits are imposed. Only upper price limits delayed the process of price discovery.

However, there are still many problems and improvements that this article does not address in terms of model building. Most price limits are caused by significant changes in a stock’s fundamental value, whereas this model functions only from the perspective of trading mechanisms. This model cannot simulate investor sentiment and order submission strategies before stocks hit price limits in the process of order formation; therefore, many miniscule phenomena of the actual market cannot be simulated. Future research could add variables that measure agent sentiment and learning mechanisms.

## Supporting Information

S1 File Figures regarding the data analysis. Figure A: The autocorrelation function graph for the yield of China Television Media. Figure B: The autocorrelation function graph for the yield of Dongfeng Electronic Technology. Figure C: The autocorrelation function graph for the yield of Shanghai Potevio. Figure D: The autocorrelation function graph for the yield of CDA. Figure E: The partial autocorrelation function graph for China Television Media. Figure F: The partial autocorrelation function graph for Dongfeng Electronic Technology. Figure G: The partial autocorrelation function graph for Shanghai Potevio. Figure H: The partial autocorrelation function graph for CDA.
(RAR)

S2 File Dataset used in the paper. The calibration data is the 5-minute trading data regarding China Television Media, Dongfeng Electronic Technology, and Shanghai Potevio. The file named ‘simulation data’ contains the simulation data simulated by our model.
(RAR)

S3 File Supplement of the simulation result. To investigate whether the conclusion depend on the parameter values, we conduct a series of additional experiment by setting different parameter values. And the file is the simulation result.
(RAR)
